# A Medical Assistive Robot for Telehealth Care During the COVID-19 Pandemic: Development and Usability Study in an Isolation Ward

**DOI:** 10.2196/42870

**Published:** 2023-04-20

**Authors:** Ruohan Wang, Honghao Lv, Zhangli Lu, Xiaoyan Huang, Haiteng Wu, Junjie Xiong, Geng Yang

**Affiliations:** 1 State Key Laboratory of Fluid Power & Mechatronic Systems School of Mechanical Engineering Zhejiang University Hangzhou China; 2 College of Electrical Engineering Zhejiang University Hangzhou China; 3 Hangzhou Shenhao Technology Co, Ltd Hangzhou China; 4 Zhejiang Key Laboratory of Intelligent Operation and Maintenance Robot Hangzhou China

**Keywords:** COVID-19, MAR, telehealth care, video chat system, mental health care

## Abstract

**Background:**

The COVID-19 pandemic is affecting the mental and emotional well-being of patients, family members, and health care workers. Patients in the isolation ward may have psychological problems due to long-term hospitalization, the development of the epidemic, and the inability to see their families. A medical assistive robot (MAR), acting as an intermediary of communication, can be deployed to address these mental pressures.

**Objective:**

CareDo, a MAR with telepresence and teleoperation functions, was developed in this work for remote health care. The aim of this study was to investigate its practical performance in the isolation ward during the pandemic.

**Methods:**

Two systems were integrated into the CareDo robot. For the telepresence system, a web real-time communications solution is used for the multiuser chat system and a convolutional neural network is used for expression recognition. For the teleoperation system, an incremental motion mapping method is used for operating the robot remotely. A clinical trial of this system was conducted at First Affiliated Hospital, Zhejiang University.

**Results:**

During the clinical trials, tasks such as video chatting, emotion detection, and medical supplies delivery were performed via the CareDo robot. Seven voice commands were set for performing system wakeup, video chatting, and system exiting. Durations from 1 to 3 seconds of common commands were set to improve voice command detection. The facial expression was recorded 152 times for a patient in 1 day for the psychological intervention. The recognition accuracy reached 95% and 92.8% for happy and neutral expressions, respectively.

**Conclusions:**

Patients and health care workers can use this MAR in the isolation ward for telehealth care during the COVID-19 pandemic. This can be a useful approach to break the chains of virus transmission and can also be an effective way to conduct remote psychological intervention.

## Introduction

### Background

The COVID-19 pandemic has been affecting the global population for more than 2 years since the World Health Organization’s declaration of its outbreak on March 11, 2020 [1]. Despite being first and foremost a health crisis, COVID-19 has the seeds of a mental health crisis [2,3]. People feel frustrated, worried, and stressed, not only due to the immediate health impacts of the virus but also due to the lack of social communication caused by movement restrictions [4-7]. In face of the pandemic, one major solution to reduce the spread of the virus is keeping social distance [1,8], which means less physical contact and even physical isolation. The era of smart medicine, known as Healthcare 4.0, makes medical care more efficient and intelligent. Healthcare 4.0 is leading to a revolution in health care services to cope with global medical challenges, especially in isolation care, in which telehealth assistance can be deployed [9,10]. Telehealth assistance allows health care workers to implement medical treatment without contact with patients, directly breaking the transmission chains of the virus [11-13]. One of the paradigm shifts in telehealth is the communication model from direct consultation to human-computer contact, in which a medical assistive robot (MAR) can be adopted as a critical way for delivering clinical mental health care to relax nervous individuals during this crisis [14,15].

Prior to the COVID-19 pandemic, the most important application of MARs was robotic surgery [16]. During the pandemic, MARs have proliferated for contactless medical care purposes. Organizations such as the World Health Organization and the Centers for Disease Control and Prevention utilize MARs for suggestion-giving, emotion-guiding, and information-sharing applications [17,18]. Moreover, to achieve a more intimate interaction, the appearance of MARs is developing toward humanoid robots. Teleoperation and telepresence functions are also integrated into the robots, allowing them to move around and monitor the patient in the isolation ward [19]. MARs, if effectively designed and used, can bridge the gap between patients and telehealth care providers during the pandemic.

**Figure 1:**
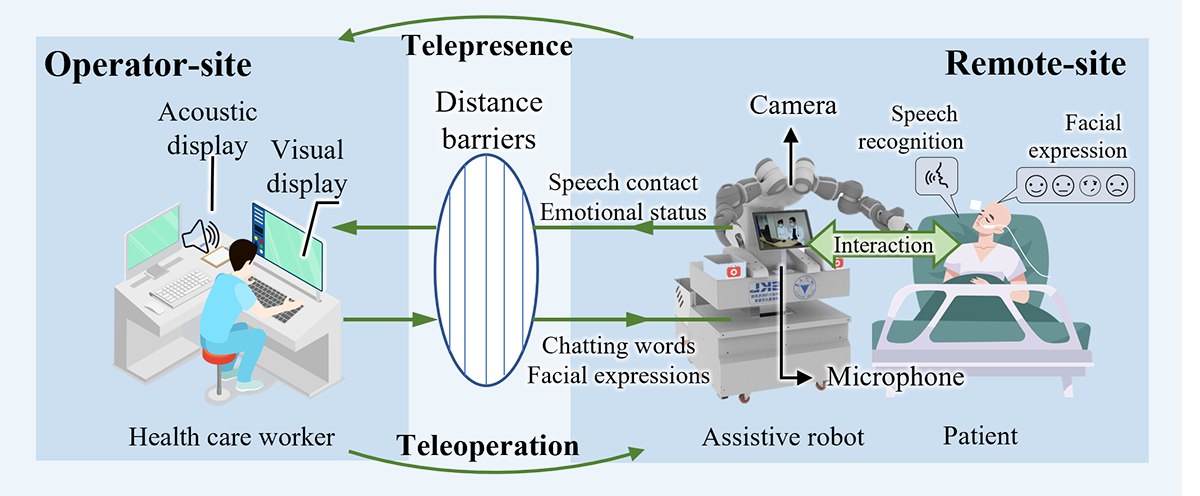
Overall architecture of the proposed medical assistive chatbot system for telepresence and telehealth care.

This paper describes a robot named CareDo with remote chatting, facial expression recognition, and teleoperation functions for telehealth care in the isolation ward, aiming to provide a safe and efficient interaction between patients and doctors during the pandemic. Figure 1 shows the overall structure of the proposed CareDo robot system. The robot is equipped with a moveable chassis, a collaborative robot (YuMi), a camera for facial expression recognition, a microphone for voice input, and a customized tablet for video chat. Both the patient and robot are in the isolation ward, and the patient can use voice commands to wake up the video chat system equipped on the robot. Once other users log in to the system, they can conduct multiuser real-time video conversations.

The primary contributions and novelties of this work are as follows: (1) an advanced MAR was developed and integrated with voice command interaction and human motion–based teleoperation; (2) a multiuser video chat system based on web real-time communication (WebRTC) was deployed with the facial expression recognition system using a trained convolutional neural network (CNN) model; and (3) a voice activation detection algorithm was designed and used during the voice command interaction, which is self-adaptive to the environment sound intensity and significantly improved voice recognition accuracy.

### Related Works

The past 2 years have witnessed an increasing number of robots in hospitals. Robots are considered to be an effective tool for cutting off the transmission of the virus.

**Figure 2:**
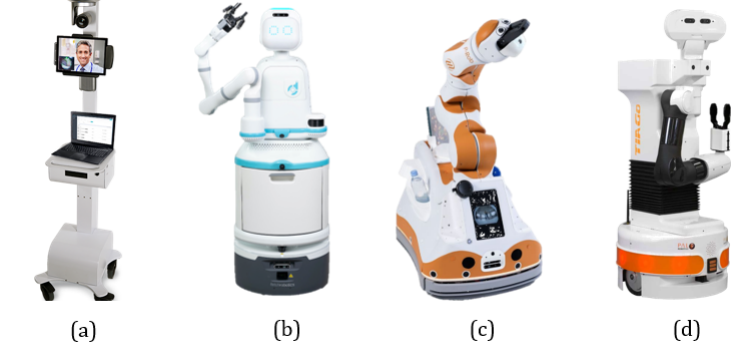
Representative medical robots used in hospitals during the COVID-19 pandemic. (a) Vici robot developed by InTouch Health (United States. (b) Moxi robot created by Diligent Robotics (United States). (c) Lio-A robot from F&P Robotics AG (Switzerland). (d) TIA-Go robot from PAL Robotics (Spain).

Various robotic solutions have been implemented for reducing unnecessary physical contacts in coronavirus management. Representative robots used for these purposes are presented in Figure 2.

A new hospital in Wuhan, China, adopted robots to deliver food, drinks, and drugs to patients in the initial stage of the COVID-19 epidemic [20]. Some of these robots are humanoid with wheeled bases and move semiautonomously in the hospital controlled by the medical staff. TIAGo [21,22], a robot operating system (ROS)-based robot platform, can perform both grasping tasks and disinfection tasks automatically. Users can choose the operating model for two scenarios through web graphical user interfaces (GUIs). Moxi [23], a robot with similar functions as the TIAGo robot, can perform repetitive chores such as grasping, pulling, opening, and guiding objects for hospital staff. Similar to the two robots described above, Lio-A also has a single arm and is able to move autonomously [24]. Moreover, Lio-A, equipped with loudspeakers and a multidirectional microphone, can understand some commands and interact with humans. Lio-A has a display screen on its front, which can show the text during its voice interaction with a human.

The assistive robots mentioned above are mainly used for logistics and disinfection. To provide emotional care, tools with human-machine interaction capacity are being released. For instance, Podrazhansky et al [25] developed a system for conducting surveys and retrieving health data. El Hefny et al [26] proposed a character-based virtual robot for reducing the risk of misinformation amplification. Amer et al [27] presented a chatbot system that can answer questions related to COVID-19. These human-machine interaction systems might lack human empathy [26]. Hence, a chatbot specially designed as a humanoid model has been proposed to improve the above systems [28]. Vici is a robot located in a hospital for telehealth [29]. Doctors can communicate with patients using the diagnostic function on Vici without direct patient contact. Pudu is a social robot for communication and telepresence functions, which can be remotely controlled using its teleoperation mode [30]. Medbot delivers telehealth in India by answering patients’ questions about health care, including home remedies, local food diets, and the detection of common diseases [31].

From these current related works, it can be seen that MARs are becoming ubiquitous, especially during the pandemic when people’s movement has been restricted. Nevertheless, a previous study showed that there are potential safety issues when using conversational assistants for health information purposes [32]. According to the benefits and drawbacks of the above accomplishments, this study considered the needs of patients, health care workers, and various application scenarios during the COVID-19 pandemic in designing the CareDo MAR.

## Methods

### System Architecture

The CareDo MAR used in this work includes two main functional parts: a telepresence system and a teleoperation system. The telepresence system contains two subsystems: a multiuser video chat system and a facial expression recognition system. The former allows the patient to talk with doctors or families without physical contact, whereas the latter can be used for patient emotional monitoring. The teleoperation system is a supported physical assistance solution for noncontact telehealth care. In the teleoperation system, the main technology is the motion mapping method, which was introduced previously [33]. With the two functional parts mentioned above, this assistive robot can be regarded as the second body of medical staff. Hence, CareDo incorporates relevant methods of a telepresence system and its novel application strategies in assisting with a teleoperation system.

**Figure 3:**
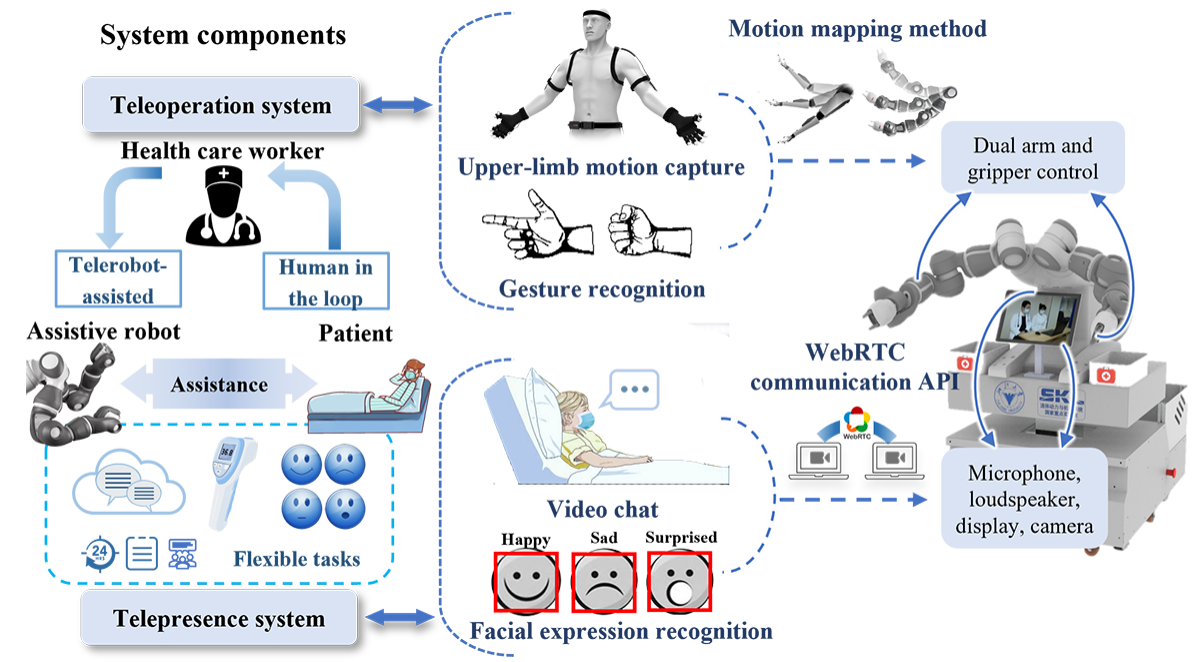
Detailed teleoperation and telepresence systems diagram of the proposed medical assistive chatbot. API: application programming interface; WebRCT: web real-time communication.

As shown in the schematic diagram of the system in Figure 3, three elements, the doctor/health care worker, the patient, and the robot, are involved. The MAR, acting as a telehealth care task performer in this system, is located in the isolation ward and controlled by the health care worker in the call center of the hospital. In this way, physical contact between the patient and medical staff is blocked. The two systems equipped on the robot play an important role in the enhanced interaction between the doctor and patient. From the site of the health care workers, the patient can receive assistive behavior, traditionally completed through psychological intervention and physical assistance, based on the teleoperation system. In addition, vital signs and the emotional status of the patient can be obtained by the doctors via the telepresence system. The dual-arm robot YuMi was chosen as the manipulator for carrying out physical assistance for the patients [34]. This system is a multinode distributed control system based on an ROS. The details are provided in the following sections.

### WebRTC-Based Video Chat System

A self-developed video chat system is integrated on the dual-arm MAR for chatting function realization. Two main technologies, a real-time speech recognition function and a noncontact telepresence GUI, are used on the video chat system of the robot. With these technologies, the robot can act as a medium for remote consultations and video chats.

For the real-time speech recognition component, the chat system is designed to recognize the voice input of a video opening construction. Toward this end, pocketsphinx, an offline voice recognition package with a specific speech recognition acoustic model, is integrated in the robot to handle the voice input. The voice activation detection algorithm is used to enable the robot to start sound recording and the recording ends with the last word. To extract voice information from the audio information, a threshold-based decision criterion is used. When the surrounding sound is stable, it has a sound energy denoted as *E.* The threshold value *ε* is then obtained using a previously described data preprocessing method [35]. The threshold *ε* represents the voice energy needed to trigger the voice recording process. In an isolation ward, the level of noise typically fluctuates because of the operation of various medical instruments. Hence, the trigger threshold *ε* was set to be self-adaptive to the environment sound intensity. Assuming *ε=f*(*E*), where *ε* ∈ {*E_min_, E_max_*}, through data set preprocessing, the threshold values *ε_min_* and *ε_max_* can be set using the sound energy *E_min_* and *E_max_*, respectively. The self-adaptive threshold value can then be expressed as:




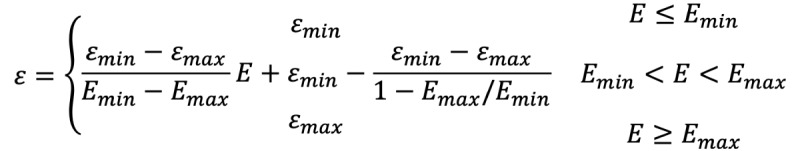




In the sound recognizing and matching process, a pretrained dictionary file is used to save the related words about logging the live video chat GUI. Then, the pocketsphinx package will find the parameter with the most similar meaning and obtain the final recognition results to determine whether to open the GUI by comparing the input voice signal and characteristic parameter in the template library.

The noncontact telepresence GUI was designed to offer a multiperson remote video platform for patient condition consulting and chatting. Therefore, the WebRTC communication technology [36] was used on the robot chat system to realize the transmission of video/audio streams. WebRTC allows network sites to establish peer-to-peer connections between browsers without intermediate media. Moreover, to go beyond a simple one-to-one video call, multiple RTCPeerConnetctions are used on WebRTC to offer connections for every endpoint to every other endpoint in a mesh configuration.

**Figure 4:**
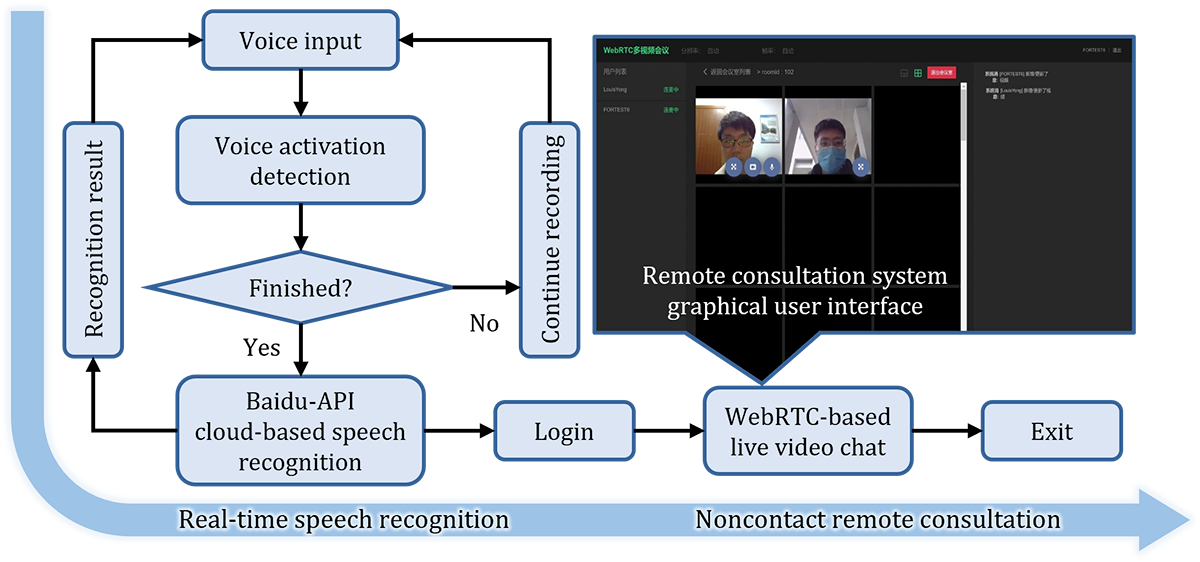
Flow-process diagram of the audio and video system for remote consultation. API: application programming interface; WebRCT: web real-time communication.

The entire video chat system structure is schematically presented in Figure 4. The system uses the voice input method mentioned above to extract the human voice from environment noise and to detect whether people have finished speaking. The most frequently used voice commands designed for the current use cases in the hospital are listed in Table 1. For all commands, 2 to 4 keywords of each chatting stage were set up to improve the reliability of speech recognition. In practical usage, the chat system of CareDo has the ability to distinguish the patient’s voice commands for contacting different doctors. Various approaches were utilized to achieve this function: (1) information of the related doctors was added to a contact list inside the robot system, enabling patients to send voice commands (including the doctor’s name) to contact an appointed doctor, and (2) doctors with different responsibilities were assigned unique numbers so that the patient can speak the voice commands with the doctor number and then the robot can directly contact the responsible doctor. In addition, as shown in Table 1, combined with a duration varying from 1 to 3 seconds of each common command, the voice activation detection algorithm is optimized and improved for enhancing the sensitivity of voice command detection. Once speaking is finished, the voice will use the online Baidu application programming interface for recognition. On the one side, the recognition results will trace back to the local computer, whereas on the other side, the voice constructions enter into the WebRTC Video & Audio System on which the consultation system GUI is built. Health care workers or families can take video calls with the patient through the GUI remotely to consult on the patient’s physical and mental health.

**Table 1:** Customized voice commands for the designed web real-time communication–based video chat system.

Chat stage and command instructions		Approximate duration (seconds)	Keywords
**Wakeup system**			
	“Hi, CareDo”	1	Hi; Hey; Hello; CareDo
	“Start remote consultation”	3	Start; Consultation
**Video chat**			
	“Create a meeting room”	3	Create; set
	“Enter the meeting room”	2	Enter
	“Call Doctor Wang”	2	Call; Doctor
**Quit system**			
	“Exit meeting room”	3	Exit; Quit
	“Thanks, CareDo”	2	Thanks; CareDo

The proposed system can realize remote consultation as well as daily family chats without health care workers entering the isolation ward. Users can log in to this video chat system through general desktop browsers such as Google Chrome and Microsoft Edge. They do not need to download specific software. Therefore, this system is safe because of reduced exposure to any vulnerabilities that may exist on the vendor’s client.

### CNN-Based Facial Expression Recognition 

**Figure 5:**
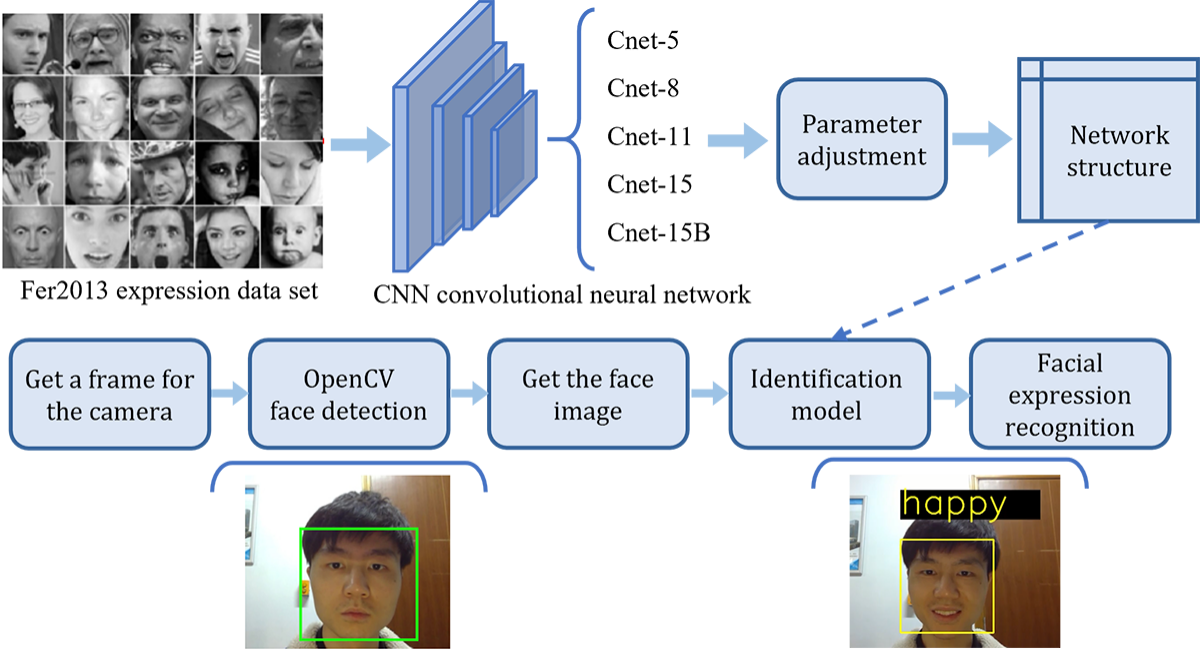
Instruction for the facial expression recognition process.

In addition to the remote video chat system, a facial expression recognition system based on CNN is used to monitor the emotional fluctuation of the patient, providing retraceable historical data for intervention therapy and promoting patients’ mental health as well as disease management. This system was achieved from our previous work on facial expression recognition for human-robot interactions [37]. Figure 5 shows the process of facial expression recognition. The source images for recognition are provided by the camera mounted on the robot. Since the source image contains some nonfacial regions, the face detection algorithm is used for detecting the region of the human face. Because of the differences in the size, aspect ratio, and illumination conditions of images, facial image preprocessing needs to be implemented to unify these image features. Measures such as image cropping, resizing, and normalizing are used to preprocess the image to remove some irrelevant information of the face region, distinguish more subtle facial information, and adjust the image size. Furthermore, random flip technology is used for removing high-frequency noise and insuring a similar distribution of the image pixels. Following image preprocessing, the CNN-based network is used for facial expression decoupling. The generative and discriminative representations are learned simultaneously. A classifier was developed by training the features obtained in the last step using a machine learning algorithm. The data set Fer2013, which consists of 35,887 grayscale images of faces with emotion, was used for training the model, as shown in Figure 5. A detailed description of the model architecture was provided previously [38]. The first 32,299 images in Fer2013 were used as the training sets and the remaining 3587 images were selected as the verification sets. For model training, we used the configuration of the 50,000 training steps with a learning rate of 0.0001. Finally, the facial recognition result is obtained through the processes mentioned above. Five common facial expressions were defined and classified in this work: neutral, surprise, sad, fear, and happy.

### Human-Cyber Physical System–Based Remote Assistive Technology

#### System Structure

**Figure 6:**
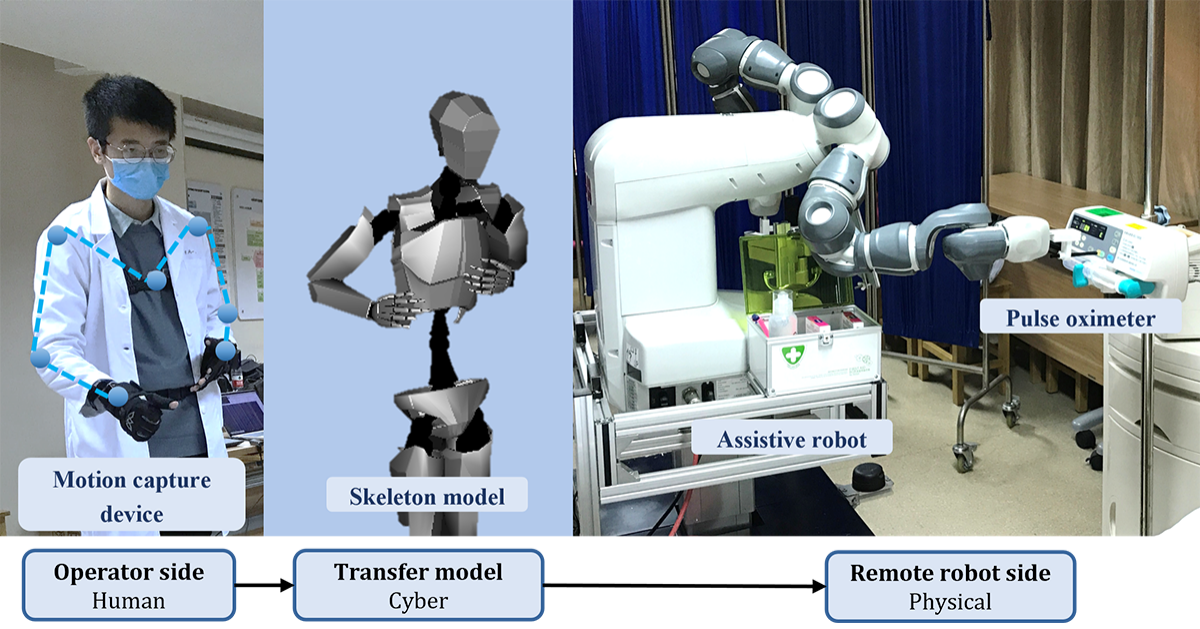
Illustration and use case of the human-cyber physical system–based remote assistive technology.

To assist patients in the isolation ward, a unique teleoperation system is proposed to provide an intuitive remote-control interface for doctors to operate the MAR. As a human-cyber physical system (HCPS)-based assistive technology, three elements are included in this system. Health care workers, as the humans in this system, wear a motion capture device suit. The MAR, as the physical entity in this system, can be remotely controlled by health care workers [38]. The cyber can be the information transferred from the human side to the robot side, where physical interventions on the patient can be implied. According to the detailed control block diagram of the system shown in Figure 6, the proposed telerobotic system can be divided into a motion-capture subsystem on the operator site and a robot-control subsystem on the robot side.

#### Human Side

The human motion capture technology is mainly used on the human side of the teleoperation system. The Perception Neuro 2.0 (PN2) motion capture suit is used to capture the real-time upper limb motion of the operator. PN2 is an adaptive motion capture device that consists of multinode inertial measurement units (IMUs), which are all located on the straps in this device [39]. IMUs can transmit the heading angle, acceleration, and angular velocity information to the hub, which is the central processing unit of PN2. However, different wearers have distinct body sizes. Therefore, to obtain the position and orientation information of the hand IMU relative to the hip IMU of each wearer, the parameters of the body parts such as arm length and shoulder width must first be measured and input into the Axis Neuron software. In addition, a self-developed executable program is used to obtain the motion tracking data from Axis Neuron, a supporting application of PN2, and communicate with the ROS. In the ROS, two nodes are established to receive and publish the motion data of the limbs and hands.

#### Robot Side

From the human side mentioned above, the position and posture data of the operator hands are obtained. Because the workspace of a human hand and the robotic manipulator is different, a previously proposed incremental pose-mapping strategy was used [33]. This method is mainly used to obtain the current human hand orientation and the increment of its position, and then to map it to the robot based on the current position of the robot. Using the open-source inverse kinetic algorithm trac_ik [40], each joint angle of the dual arm can be obtained corresponding to the current robot pose. The predefined different hand gestures stand for different robot motion control commands. Based on these, Lv et al [33] developed a hybrid mapping method of hand gestures and limb motion. Before the teleoperation begins, the operator does not need to assume the same posture as the robot arm. Hand gestures can be defined to enable and disable motion mapping. Hence, on the human side, the action of the operator can be more flexible, while on the robot side, the manipulator can reach any position in its workspace.

### Ethics Considerations

Approval of all ethical and experimental procedures and protocols was granted by the Clinical Research Ethics Committee of the First Affiliated Hospital, Zhejiang University (FAHZU; approval number IIT20200048A-R1), and the study was performed in line with the full informed consent of the volunteers, in accordance with all local laws.

## Results

### Performance of the WebRTC-Based Video Conference System

**Figure 7:**
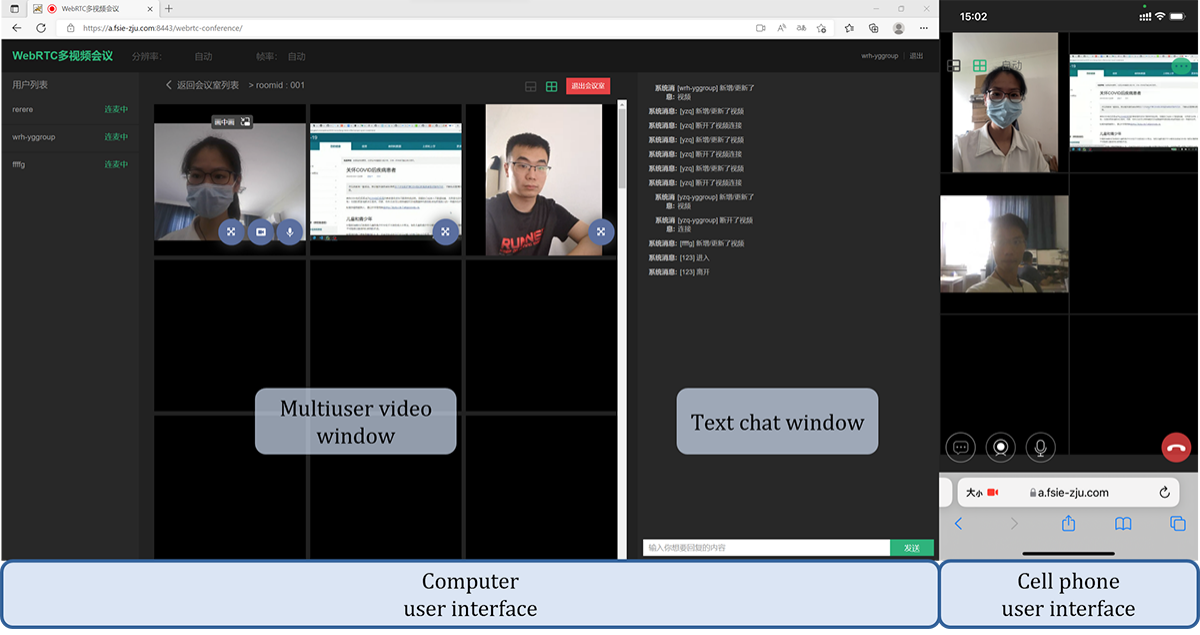
Customized graphical user interface on the CareDo robot screen and a mobile phone.

The WebRTC-based video chat system provides essential telemedicine services. Compared with other video chat systems, this system adopts peer-to-peer connection, which is easy to manage and deploy. Figure 7 shows a practical use case of the video chat system on both a computer and a mobile phone and a video presentation of this use case is provided in Multimedia Appendix 1.

The GUI on the browser is used as shown in the window on the left side of Figure 7. Integrated with the speech recognition function, the video chat system can be awakened and controlled by the voice command from the user, both from the patient side and the remote doctor side. This technique enables the noncontact interaction between the robot and patients, which decreases the cross-infection risks for doctors and other medical staff when they operate the robots. In this test case, three subjects were in different locations and used different local area networks to log in to the system at the same time. Two subjects entered the system by using the voice wakeup function and they then launched the video and voice applications for communication. One subject opened the remote screen through which the medical instructions or psychological counseling methods were shared. The text window chat function was tested for transferring text messages and medical documents. This GUI was tested on both a computer and a mobile phone. The tests confirmed its usability in this video chat system.

### Facial Expression Recognition Performance

The facial expression recognition performance was evaluated during clinical trials at the FAHZU. Using the camera integrated on the front screen of the CareDo robot, the facial expressions of the patients were recorded and analyzed. The facial expression recognition data set was collected from 12 subjects, including 6 female and 6 male subjects, ranging in age from 15 to 60 years and evenly distributed from three groups: the young group (15-25 years old), middle-aged group (25-45 years old), and older adult group (45-60 years old). The facial expression recognition system worked 8 hours a day and each subject’s facial expression was recorded 15 times. The final data set was composed of 180 records (12 subjects×15 times/subject), among which 152 valid records were obtained. Table 2 shows the verification results of the facial expression recognition of one patient in a single day using the CareDo robot in the isolation ward. Cross-validation was conducted for the recorded expressions of the patients and the recognition accuracy is provided in the table for all five expression types. From the validation results, we can easily see that the neutral facial expression was detected as the most common emotion for this test, followed by the happy facial expression. The highest recognition accuracy reached 95% for the happy expression.

**Table 2:** Accuracy of recognition of patient facial expressions.

Facial expressions	Records in one day (times)	Recognition accuracy, %
Surprise	0	Not applicable
Sad	5	80.0
Neutral	125	92.8
Fear	2	50.0
Happy	20	95.0

### Verification in the FAHZU Emergency Intensive Care Unit

**Figure 8:**
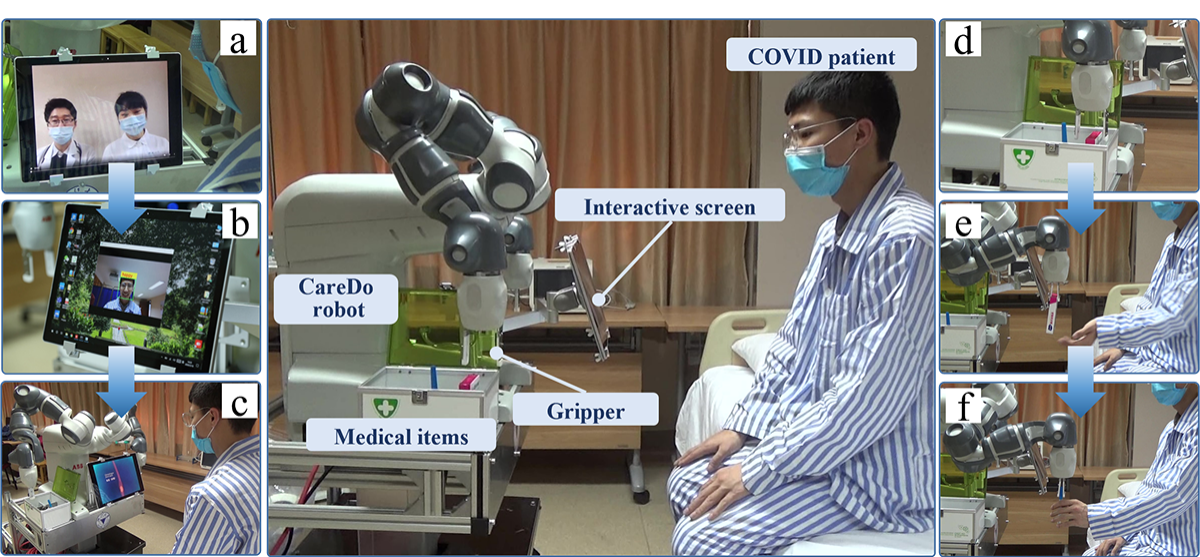
Application cases of the CareDo assistive robot used in the First Affiliated Hospital, Zhejiang University emergency intensive care unit during the COVID-19 pandemic. (a) Voice and video interaction between the doctor and patient. (b) Facial expression recognition. (c) Educational videos to provide information to the patient. (d-f) Remote medical delivery tasks delivered via teleoperation.

According to the current diagnosis and treatment operation requirements of the isolation ward for COVID-19 patients, we developed a new type of the CareDo MAR to assist in the diagnosis and treatment operations of medical staff. After validation in a laboratory environment, the robot was checked by the clinical research ethics committee of the FAHZU and obtained investigator-initiated trial (IIT) ethics approval. The CareDo robot was then applied in the emergency intensive care unit (EICU) of the FAHZU for preliminary clinical function verification, as shown in Figure 8.

Aiming at reducing the risk of infection to health care staff due to exposure to the COVID-19 virus, the robot was used in the isolation ward to perform remote care tasks through teleoperation. For the WebRTC-based video chat system, the COVID-19 patient interacted with the remote doctors using the interactive screen on the front of the robot. As shown in Figure 8a, using voice and video interactive devices, doctors can chat with the patient and perform some routine diagnoses remotely. In addition, the mental health status of the quarantined patients in the isolation ward would be a greater concern than that of general patients. Hence, with use of the facial expression recognition system, the CareDo robot acts as the bedside companion of the patient by observing the patient’s facial expression status, as shown in Figure 8b. The doctor can then communicate with the patient remotely to provide any psychological intervention guidance according to the results and analysis of facial expression recognition. The interactive screen can also play some related informational and educational videos for patients (Figure 8c). For the remote assistive system, the CareDo robot was teleoperated to perform some medical delivery tasks using the proposed HCPS-based remote assistive technology, as shown in Figure 8d-f. The robot in the teleoperation function can be used for delivering medicine or medical supplies such as a thermometer, food, personal supplies, and other required items to patients. Other details about the implementation of the MAR in the FAHZU were reported in our previous paper [13]. In summary, the developed CareDo robot has been applied in real isolation wards with the video chat system and the remote assistive system. All of the desired functions have been preliminarily achieved based on the basic requirements of both doctors and patients, and positive feedback from the users has been reported in the real clinical trials in the FAHZU.

## Discussion

### Improvement of Telehealth Services

The emergence of COVID-19 has brought great changes to the medical industry, especially the telemedicine service. The CareDo robot offers another new form of telehealth assistance. In this work, an advanced telerobotic system was developed. Its efficient deployment in hospitals was applied by leveraging the enabling technologies of Healthcare 4.0. Techniques, including high-performance wireless communications, high-quality remote audio and video systems, an intelligent remote-controlled robot, and wearable sensors for motion capture, are used to assist and protect health care professionals.

With these functions, CareDo can execute relevant operations of a remote video system according to the patient’s voice instruction, monitor patients’ mental health status, and grasp and deliver medical supplies through teleoperation. During the utilization period in a hospital, CareDo can mitigate the risk of nosocomial infection and therefore contribute to accelerated recovery of the COVID-19 epidemic.

In the proposed telerobotic system, telemedical staff can use remote video to talk with patients and remotely operate the robot outside the negative pressure ward to complete nursing work, avoiding cross-infection caused by their frequent close contact with patients. The proposed system can realize the real-time monitoring and recording of patients’ emotional changes, providing retraceable historical data for intervention therapy and promoting patients’ mental health as well as disease management. The system makes significant contributions to the mitigation and suppression of COVID-19 transmission chains for impacted societies.

### Limitations and Future Work

This effort offers a quick solution of remote video and dialogue between patients and doctors during the pandemic. However, several limitations still exist. First, the user experience has not been deeply investigated or estimated during the use of a single function such as a video chat. Second, for the telepresence system, the recognition accuracy of facial expressions such as sad and fear still need to be optimized. A longer patient usage time is suggested to obtain more samples and records. During the implementation and clinical trials, the influence of wearing a mask was not considered or tested in this work. Wearing a mask will cover the lower part of the face and make most facial features invisible, which will decrease the facial expression recognition accuracy [41,42]. Third, for the teleoperation system, this work was based on unilateral teleoperation and we did not investigate the force feedback from the robot to the operator. Furthermore, the dual arms are controlled by human hands, lacking consideration of cooperation tasks. More complex tasks and flexible control methods should be considered to achieve compliance control.

Future work could focus on the improvement of functionality and integration. Based on the exploitable functions of WebRTC, the facial expression recognition function can be integrated into the real-time communication system. The influence of wearing masks on facial expression recognition will be considered and investigated in the future. The user operation process can be simplified while the security of the remote video chat system can be evaluated. Further study can also focus on developing cost-effective MARs for applications in more generalized telehealth care scenarios.

### Conclusions

This article described the design and development of CareDo, a MAR devised to provide telehealth care to COVID-19 patients in the isolation ward. Three key technologies used on this robot are (1) a telepresence system in which the user can log in with voice input, enabling patients, doctors, and patients’ family members to have safe and real-time remote chats; (2) a facial expression recognition system that can monitor the patients’ emotional fluctuations; and (3) multinode ROS–based teleoperation technology that assists the robot in the isolation ward to perform other tasks such as delivering medical supplies. The CareDo robot was used in the EICU of the FAHZU for function verification under IIT ethics approval. The results showed that use of this MAR in the hospital can reduce the risk of cross-infection between patients and doctors. Moreover, the multiuser video chat system allows patients to talk with doctors and their family, which can relieve the patients’ mental stress from isolation.

## Appendix

Multimedia Appendix 1 Experiment and verification in the hospital.

## Abbreviations

CNN: convolutional neural network
EICU: emergency intensive care unit
FAHZU: First Affiliated Hospital, Zhejiang University
GUI: graphical user interface
HCPS: human-cyber physical system
IIT: investigator-initialed trial
IMU: inertial measurement unit
MAR: medical assistive robot
PN2: Perception Neuron 2
ROS: robot operating system
WebRTC: web real-time communications
